# Effectiveness of one-shot dual-energy subtraction chest radiography with flat-panel detector in distinguishing between calcified and non-calcified nodules

**DOI:** 10.1038/s41598-023-36785-y

**Published:** 2023-06-12

**Authors:** Kojiro Minato, Motohiko Yamazaki, Takuya Yagi, Tetsuhiro Hirata, Masaki Tominaga, Kyoryoku You, Hiroyuki Ishikawa

**Affiliations:** Department of Radiology and Radiation Oncology, Niigata University Graduate School of Medical and Dental Sciences, Niigata, Japan

**Keywords:** Image processing, Cancer imaging

## Abstract

The purpose of this study was to evaluate the added value of the soft tissue image obtained by the one-shot dual-energy subtraction (DES) method using a flat-panel detector compared with the standard image alone in distinguishing calcified from non-calcified nodules on chest radiographs. We evaluated 155 nodules (48 calcified and 107 non-calcified) in 139 patients. Five radiologists (readers 1 − 5) with 26, 14, 8, 6 and 3 years of experience, respectively, evaluated whether the nodules were calcified using chest radiography. CT was used as the gold standard of calcification and non-calcification. Accuracy and area under the receiver operating characteristic curve (AUC) were compared between analyses with and without soft tissue images. The misdiagnosis ratio (false positive plus false negative ratios) when nodules and bones overlapped was also examined. The accuracy of all radiologists increased after adding soft tissue images (readers 1 − 5: 89.7% vs. 92.3% [P = 0.206], 83.2% vs. 87.7% [P = 0.178], 79.4% vs. 92.3% [P < 0.001], 77.4% vs. 87.1% [P = 0.007], and 63.2% vs. 83.2% [P < 0.001], respectively). AUCs for all the readers improved, except for reader 2 (readers 1 − 5: 0.927 vs. 0.937 [P = 0.495], 0.853 vs. 0.834 [P = 0.624], 0.825 vs. 0.878 [P = 0.151], 0.808 vs. 0.896 [P < 0.001], and 0.694 vs. 0.846 [P < 0.001], respectively). The misdiagnosis ratio for nodules that overlapped with the bone decreased after adding soft tissue images in all readers (11.5% vs. 7.6% [P = 0.096], 17.6% vs. 12.2% [P = 0.144], 21.4% vs. 7.6% [P < 0.001], 22.1% vs. 14.5% [P = 0.050] and 35.9% vs. 16.0% [P < 0.001], respectively), particularly that of readers 3 − 5. In conclusion, the soft tissue images obtained using one-shot DES with a flat-panel detector have added value in distinguishing calcified from non-calcified nodules on chest radiographs, especially for less experienced radiologists.

## Introduction

Calcification is one of the most reliable indicators of the benign status of solitary pulmonary nodules. However, the accuracy of detecting calcification using chest radiography is low. Berger et al*.*^1^ reported that the sensitivity and specificity of chest radiography in detecting calcification are 0.50 and 0.87, respectively.

Dual-energy subtraction (DES) chest radiograph is able to generate soft tissue images without bony and calcified structures as well as standard images^2,3^. If a nodule seen on a standard image is disappeared on a soft tissue image, it should be calcified^2^. In contrast, non-calcified nodules are detected on both standard and soft tissue images. Therefore, soft tissue images obtained from DES may help in differentiating calcified and non-calcified nodules. There are two types of DES: one-shot (single-exposure) and dual-shot (dual-exposure) systems. However, dual-shot DES is not widely used because of the high radiation dose and motion artefacts caused by the body movement between the first and second shots^2^. One-shot DES solves these problems by acquiring both images without motion artefacts in a single shot. Ishigaki et al. used one-shot DES with computed radiography and have shown its superiority compared to standard chest images in detecting pulmonary nodules, calcification in a nodule, and rib lesions^4^. Recently, traditional one-shot DES using film-screen radiography or computed radiography has developed into one-shot DES using a flat-panel detector^2^.

Although the usefulness of DES has already been reported on chest radiography, most studies have not evaluated distinguishability between calcified and non-calcified nodules, but rather evaluated nodule detectability^2,3,5–10^. In addition, most studies have used two-shot DES^3,6,9,10^ or have used one-shot DES by means of film-screen radiography or computed radiography instead of flat-panel detectors^4,5,8,11^. Therefore, the diagnostic performance of one-shot DES with a flat-panel detector for calcified nodules is not well established. Compared with film-screen radiography and computed radiography, flat-panel detectors improve the image quality and reduce radiation doses^12^.

This study aimed to evaluate the added value of the soft tissue image obtained using one-shot DES with a flat-panel detector compared with the standard image alone in distinguishing calcified from non-calcified nodules on chest radiographs.

## Results

### CT findings of nodules

CT evaluation for 155 nodules included in this study is shown in Table 1. Of the calcified nodules, 16 nodules were in the right lung (9 upper, 2 middle, and 5 lower lobes), and 14 were in the left lung (8 upper and 6 lower lobes). Of the non-calcified nodules, 59 nodules were in the right lung (32 upper, 7 middle, and 20 lower lobes), and 43 were in the left lung (29 upper and 14 lower lobes). The remaining 18 calcified and 5 non-calcified nodules were present on the thoracic wall or skin. The patterns of calcification for calcified nodules were as follows: 41 diffuse, 5 central, 1 laminated, and 1 other type. Therefore, all except one calcified nodule showed a benign pattern of calcification (diffuse, central, or laminated)^13^. Regarding the type of non-calcified nodules, 87 were solid, and 20 were part-solid nodules. The median diameters of calcified and non-calcified nodules were 8 mm (range, 3 − 17 mm) and 14 mm (range, 5 − 29 mm), respectively. Fourteen of 20 part-solid nodules had a solid component ≥ 10 mm, and most part-solid nodules (18/20) had a percentage of solid component ≥ 50%.

**Table 1:** CT findings of nodules in this study.

Characteristics			Calcified nodules (n = 48)	Non-calcified nodules (n = 107)
Location of nodules	Right lung	Upper lobe	9	32
		Middle lobe	2	7
		Lower lobe	5	20
	Left lung	Upper lobe	8	29
		Lower lobe	6	14
	Right thoracic wall or skin^a^		9	1
	Left thoracic wall or skin^b^		9	4
Pattern of calcification	Diffuse		41	
	Central		5	
	Laminated		1	
	Popcorn		0	
	other		1	
Type of non-calcified nodules	Solid nodule			87
	Part-solid nodule			20
	Pure GGN			0
Diameter of nodules^c^	< 10 mm		29	35
	10 mm ≤		19	72
Diameter of solid component in part solid nodules	< 10 mm			6
	10 mm ≤			14
Percentage of solid component of part solid nodules^d^	< 50%			2
	50% ≤			18

*GGN* ground-glass nodule.

^a^includes 10 bony nodules, ^b^includes 9 bony nodules and 4 skin nodules, ^c^The median diameter of calcified nodules was 8 mm (range, 3 − 17 mm), and that of non-calcified nodules was 14 mm (range, 5 − 29 mm), ^d^calculated by (diameter of solid component)/(whole diameter).

### Diagnostic performance

The inter-observer agreement among the five readers improved with the addition of soft tissue images to the analysis (0.334 [fair agreement] vs. 0.688 [substantial agreement]). The results of diagnostic performance are summarized in Tables 2, 3 and 4. The sensitivity and accuracy of all radiologists were increased after adding soft tissue images (Table 2). The sensitivities of readers 1 − 5 were 68.8% vs. 77.1% (P = 0.206), 56.3% vs. 62.5% (P = 0.513), 58.3% vs. 77.1% (P = 0.020), 35.4% vs. 70.8% (P < 0.001), and 79.2% vs. 83.3% (P = 0.527), respectively. The accuracies were 89.7% vs. 92.3% (P = 0.206), 83.2% vs. 87.7% (P = 0.178), 79.4% vs. 92.3% (P < 0.001), 77.4% vs. 87.1% (P = 0.007), and 63.2% vs. 83.2% (P < 0.001), respectively. The specificity was improved after adding soft tissue images for three radiologists with 14, 8 and 3 years of experience (readers 1 − 5: 99.1% vs. 99.1% [P = 1.000], 95.3% vs. 99.1% [P = 0.103], 88.8% vs. 99.1% [P = 0.002], 96.3% vs. 94.4% [P = 0.480], and 56.1% vs. 83.2% [P < 0.001], respectively). The area under the receiver operating characteristic curves (AUCs) improved after adding soft tissue images in readers with 26, 8, 6 and 3 years of experience, and statistical significance was observed in readers with 6 and 3 years (readers 1 − 5: 0.927 vs. 0.937 [P = 0.495], 0.853 vs. 0.834 [P = 0.624], 0.825 vs. 0.878 [P = 0.151], 0.808 vs. 0.896 [P < 0.001], and 0.694 vs. 0.846 [P < 0.001], respectively) (Table 3). The diagnostic accuracies in nodule diameters < 10 mm and ≥ 10 mm are presented in Table 4. In both cases, accuracies were improved after adding soft tissue images for all readers, particularly for less experienced radiologists. Accuracies in < 10 mm for readers 1 − 5 were 89.1% vs. 90.6% (P = 0.655), 79.7% vs. 81.3% (P = 0.796), 78.1% vs. 87.5% (P = 0.058), 68.8% vs. 81.3% (P = 0.059), and 62.5% vs. 76.6% (P = 0.029), respectively. The accuracies in ≥ 10 mm were 90.1% vs. 93.4% (P = 0.180), 85.7% vs. 92.3% (P = 0.083), 80.2% vs. 95.6% (P < 0.001), 83.5% vs. 91.2% (P = 0.052), and 63.7% vs. 87.9% (P < 0.001), respectively. The accuracy in nodule diameter ≥ 10 mm was higher than that in diameter < 10 mm for all readers.

**Table 2:** Comparison of the sensitivity, specificity, and accuracy of each reader while evaluating the standard image alone vs. the standard and the soft tissue images.

Years of experience	Standard image alone			Standard and soft tissue images			P value 1	P value 2	P value 3
	Sensitivity	Specificity	Accuracy	Sensitivity	Specificity	Accuracy			
26 (reader 1)	68.8% (33/48)	99.1% (106/107)	89.7% (139/155)	77.1% (37/48)	99.1% (106/107)	92.3% (143/155)	0.206	1.000	0.206
14 (reader 2)	56.3% (27/48)	95.3% (102/107)	83.2% (129/155)	62.5% (30/48)	99.1% (106/107)	87.7% (136/155)	0.513	0.103	0.178
8 (reader 3)	58.3% (28/48)	88.8% (95/107)	79.4% (123/155)	77.1% (37/48)	99.1% (106/107)	92.3% (143/155)	0.020	0.002	< 0.001
6 (reader 4)	35.4% (17/48)	96.3% (103/107)	77.4% (120/155)	70.8% (34/48)	94.4% (101/107)	87.1% (135/155)	< 0.001	0.480	0.007
3 (reader 5)	79.2% (38/48)	56.1% (60/107)	63.2% (98/155)	83.3% (40/48)	83.2% (89/107)	83.2% (129/155)	0.527	< 0.001	< 0.001

The sensitivity and accuracy were improved after adding soft tissue images for all radiologists. The specificity was improved after adding soft tissue images for three radiologists (14, 8, and 3 years of experience). P values 1, 2, and 3 denote statistically significant differences in sensitivity, specificity, and accuracy, respectively.

**Table 3:** Comparison of the AUCs of each reader performing analysis with and without soft tissue images.

Years of experience	AUC in using standard image alone	AUC in using both standard and soft tissue images	P value
26 (reader 1)	0.927	0.937	0.495
14 (reader 2)	0.853	0.834	0.624
8 (reader 3)	0.825	0.878	0.151
6 (reader 4)	0.808	0.896	< 0.001
3 (reader 5)	0.694	0.846	< 0.001

The AUCs were improved in readers with 26, 8, 6 and 3 years of experience, with statistical significance in readers with 6 and 3 years of experience. P value denotes statistically significant differences in AUC.

*AUC* area under the receiver operating characteristic curve.

**Table 4:** Comparison of the accuracy in nodule diameter < 10 mm and 10 mm ≤ while evaluating the standard image alone vs. the standard and the soft tissue images.

Years of experience	Nodule diameter < 10 mm (n = 64)			Nodule diameter ≥ 10 mm (n = 91)		
	Standard image alone	Standard and soft tissue images	P value	Standard image alone	Standard and soft tissue images	P value
26 (reader 1)	89.1% (57/64)	90.6% (58/64)	0.655	90.1% (82/91)	93.4% (85/91)	0.180
14 (reader 2)	79.7% (51/64)	81.3% (52/64)	0.796	85.7% (78/91)	92.3% (84/91)	0.083
8 (reader 3)	78.1% (50/64)	87.5% (56/64)	0.058	80.2% (73/91)	95.6% (87/91)	< 0.001
6 (reader 4)	68.8% (44/64)	81.3% (52/64)	0.059	83.5% (76/91)	91.2% (83/91)	0.052
3 (reader 5)	62.5% (40/64)	76.6% (49/64)	0.029	63.7% (58/91)	87.9% (80/91)	< 0.001

P value denotes statistically significant differences in accuracy.

### Misdiagnosis ratio of nodules with and without bone overlap

The misdiagnosis ratios of overlapping and non-overlapping nodules with the bone are presented in Table 5. There were 131 nodules that overlapped with the bone and 24 nodules that did not. The misdiagnosis ratio for nodules that overlapped with the bone decreased after adding soft tissue images in all readers (11.5% vs. 7.6% [P = 0.096], 17.6% vs. 12.2% [P = 0.144], 21.4% vs. 7.6% [P < 0.001], 22.1% vs. 14.5% [P = 0.050], and 35.9% vs. 16.0% [P < 0.001], respectively), particularly for less experienced readers (8, 6 and 3 years). For the nodules without overlapping with the bone, misdiagnosis ratio of less experienced radiologists (8, 6 and 3 years) decreased after adding soft tissue images (readers 1 − 5: 4.2% vs. 8.3% [P = 0.317], 12.5% vs. 12.5% [P = 1.000], 16.6% vs. 8.3% [P = 0.157], 25.0% vs. 4.2% [P = 0.025], and 41.7% vs. 20.8% [P = 0.059], respectively).

**Table 5:** Comparison of the misdiagnosis ratio of each reader while evaluating the standard image with and without the soft tissue image.

Years of experience	Overlapped with bone (n = 131)			Not overlapped with bone (n = 24)		
	Standard image alone	Standard and soft tissue images	P value	Standard image alone	Standard and soft tissue images	P value
26 (reader 1)	11.5% (15/131)	7.6% (10/131)	0.096	4.2% (1/24)	8.3% (2/24)	0.317
14 (reader 2)	17.6% (23/131)	12.2% (16/131)	0.144	12.5% (3/24)	12.5% (3/24)	1.000
8 (reader 3)	21.4% (28/131)	7.6% (10/131)	< 0.001	16.6% (4/24)	8.3% (2/24)	0.157
6 (reader 4)	22.1% (29/131)	14.5% (19/131)	0.050	25.0% (6/24)	4.2% (1/24)	0.025
3 (reader 5)	35.9% (47/131)	16.0% (21/131)	< 0.001	41.7% (10/24)	20.8% (5/24)	0.059

The misdiagnosis ratio (false positive plus false negative ratios) decreased after adding soft tissue images, especially for nodules overlapped with bone. P value denotes statistically significant differences in accuracy.

### Representative images

**Figure 1:**
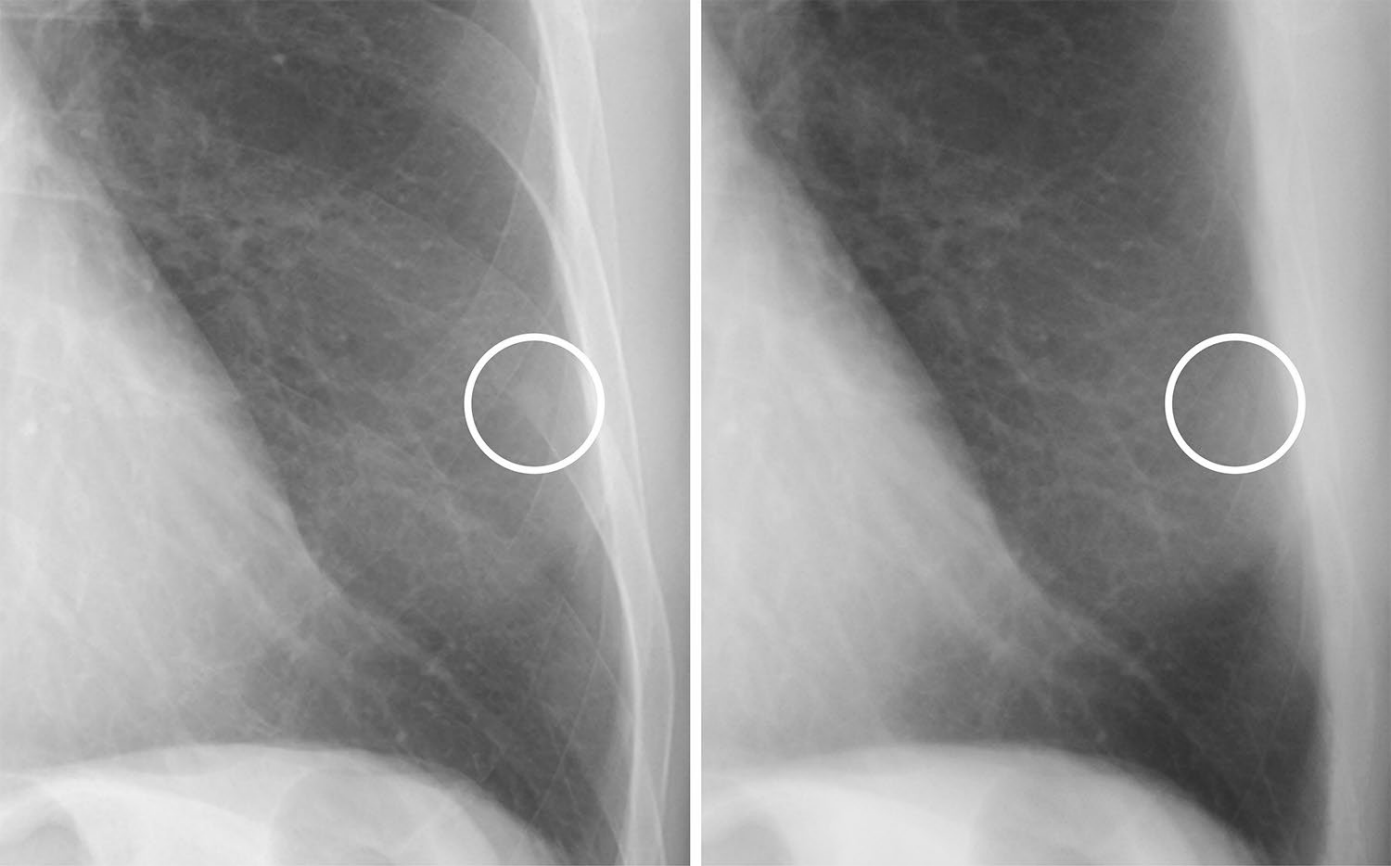
A case of a calcified nodule overlapping with the bone. (**left**) On the standard image, the evaluation of the presence or absence of calcification among readers was not consistent (confidence level of each reader: 4, 3, 3, 4 and 2, respectively). (**right**) In the soft tissue image, the nodule disappeared. All readers correctly evaluated it as calcified (confidence level of each reader: 1, 1, 2, 2 and 2, respectively).

**Figure 2:**
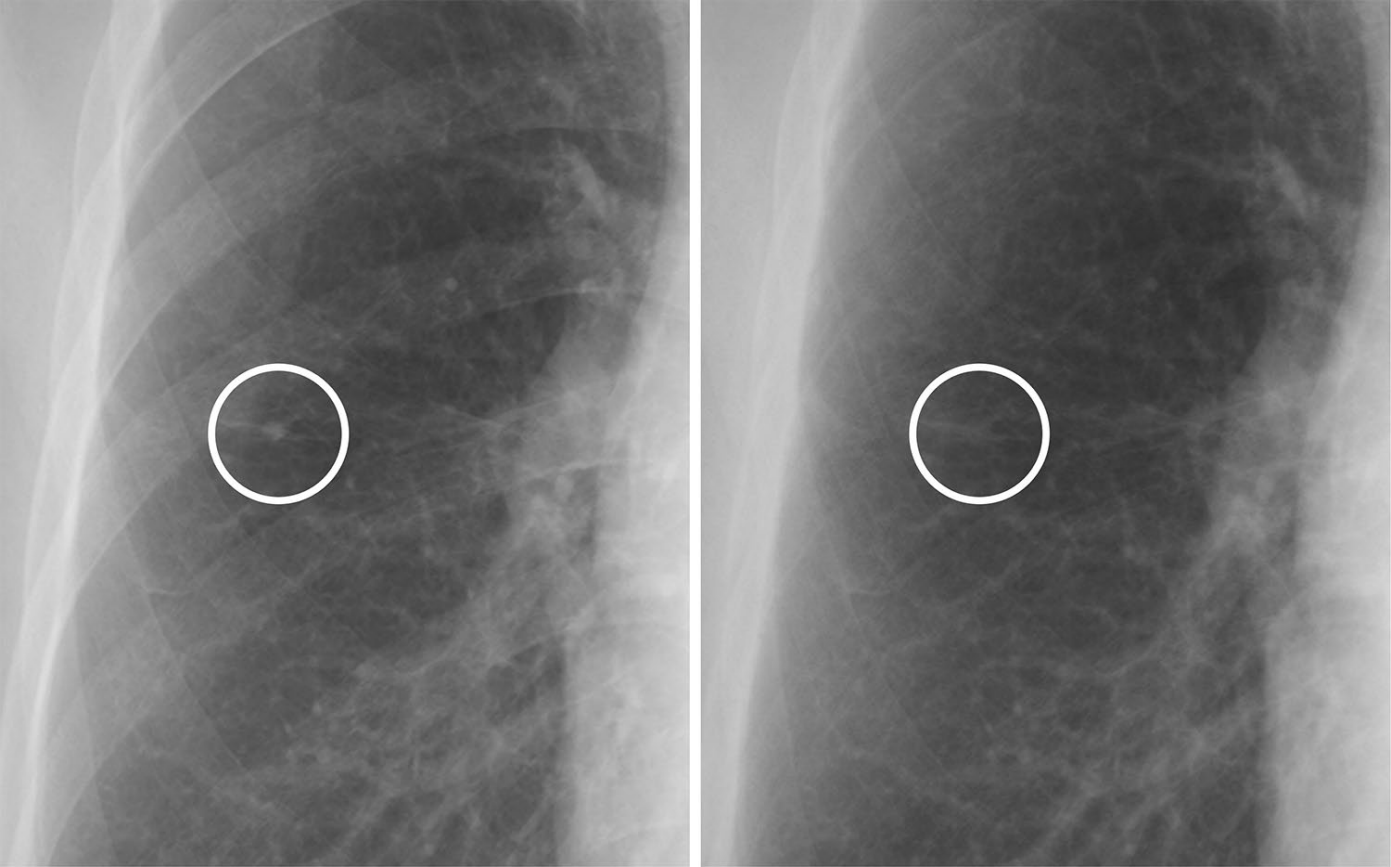
A case of a calcified nodule not overlapping with the bone. (**left**) On the standard image, the evaluation of the presence or absence of calcification among readers was not consistent (confidence level of each reader: 1, 3, 2, 3 and 2, respectively). (**right**) In the soft tissue image, the nodule disappeared. All readers correctly evaluated it as calcified (confidence level of each reader: 1, 1, 2, 1 and 2, respectively).

**Figure 3:**
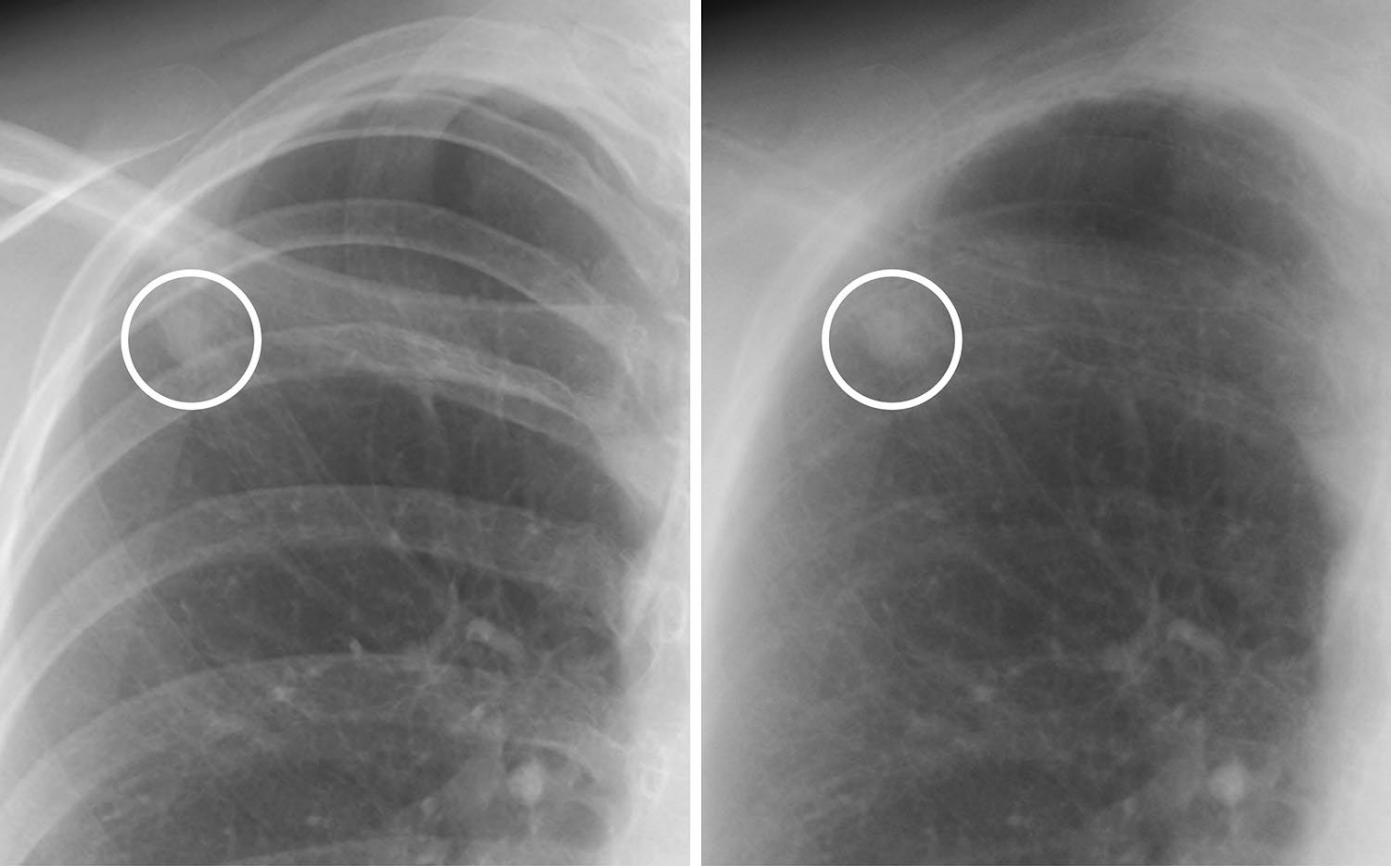
A case of a non-calcified nodule overlapping with the bone. (**left**) On the standard image, the evaluation of the presence or absence of calcification among readers was not consistent (confidence level of each reader: 3, 2, 2, 4 and 2, respectively). (**right**) In the soft tissue image, the nodule did not disappear. All readers correctly evaluated it as non-calcified (confidence level of each reader, 5, 5, 5, 5 and 4, respectively).

**Figure 4:**
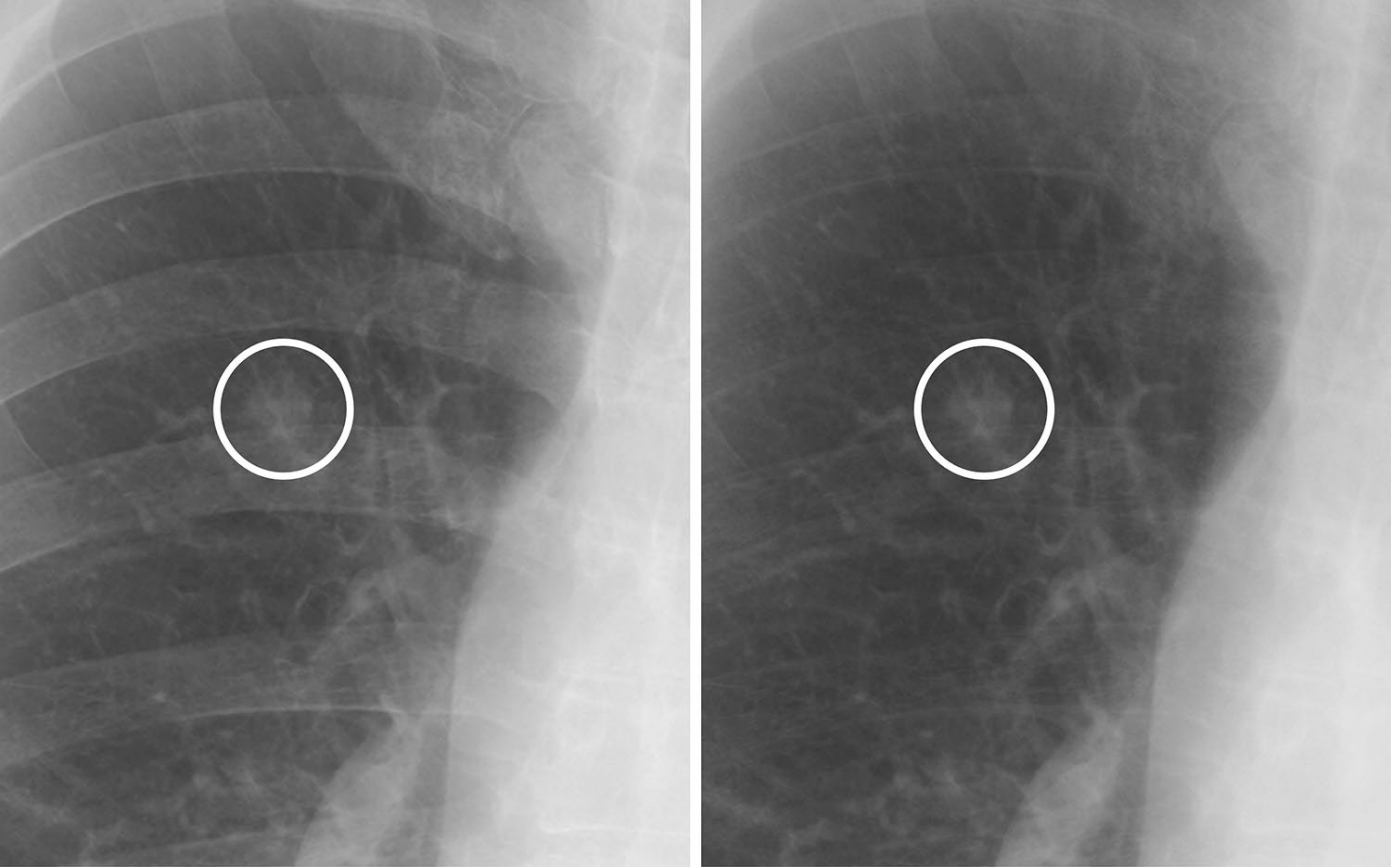
A case of a non-calcified nodule not overlapping with the bone. (**left**) On the standard image, the evaluation of the presence or absence of calcification among readers was not consistent (confidence level of each reader: 5, 5, 3, 4 and 2, respectively). (**right**) In the soft tissue image, the nodule did not disappear. All readers correctly evaluated it as non-calcified (confidence level of each reader: 5, 5, 5, 5 and 4, respectively).

Representative images of nodules that were correctly diagnosed using soft tissue images are shown in Figs. 1, 2, 3 and 4.

## Discussion

Herein, we showed that the use of standard images along with soft tissue images improved the distinguishability between calcified and non-calcified nodules, particularly for less experienced radiologists. Moreover, the combined use of the images had a higher inter-observer agreement than the standard image alone.

We also observed that misdiagnosis ratio of nodules that overlapped with the bone decreased after adding soft tissue images in all readers, whereas that of non-overlapping nodules decreased for only three readers. This implies that additional soft tissue imaging may be useful especially in nodules overlapped with the bone. Furthermore, regarding the non-overlapping nodules, the misdiagnosis ratio decreased in less experienced radiologists.

Chest radiography is still popular and frequently performed during clinical examinations. Chest radiography is widely available, has low cost, and patients undergoing chest radiography are exposed to low radiation doses. However, compared to CT, this technique has some limitations, such as lower accuracy for the detection of abnormalities^1^.

Several techniques that remove bones and calcified structures from chest radiographs have developed to improve the diagnostic performance. One is a software-based bone suppression method^5^. The second is the DES method used in our study, which removes bones by acquiring two images with different energies and then subtracting them^6,14,15^. In addition, owing to recent advances in artificial intelligence (AI), bone suppression algorithms using deep learning have also been developed^16^. Although the utility of these bone suppression methods is known, evidence regarding the superior method for detecting calcified nodules remains insufficient. Further studies comparing the diagnostic performance of different bone suppression techniques are needed.

Many studies have shown that using the DES method could improve the diagnostic performance on chest radiography^2–11^. Nevertheless, most previous studies have evaluated the detectability of the lung nodules rather than the presence or absence of nodule calcification^2,3,5–10^. In addition, most previous studies used dual-shot DES^3,6,9,10^ or used one-shot DES with film-screen radiography or computed radiography^4,5,8,11^; these studies did not use one-shot DES with a flat-panel detector. To the best of our knowledge, the present study is the first to evaluate the effectiveness of one-shot DES with a flat-panel detector to differentiate between calcified and non-calcified nodules.

One of the disadvantages of DES is the need for special equipment, which limits their widespread use. To overcome this issue, our future aim is to develop an AI that automatically generates soft tissue images from standard chest radiography. Recently, Liang et al. proposed an AI algorithm for image-to-image translation between standard and soft tissue images obtained using DES chest radiography and demonstrated its effectiveness^17^. The development of such AI algorithms will benefit from the DES system, even in institutions without special equipment.

Our study had several limitations. First, this study was retrospective performed in a single institution, and only patients who underwent both chest radiography and CT within an interval of 3 months were included. Therefore, the possibility of selection bias might not be ruled out. Second, diffuse and partially calcified nodules were not evaluated separately. It is necessary to investigate whether DES can distinguish between diffuse and partially calcified nodules in further studies because the pattern of calcification in a pulmonary nodule is related to the frequency of benignity and malignancy^13^. Third, we did not confirm whether the nodules analysed in this study were pathologically benign or malignant. Fourth, all the readers were aware of the location of the nodules on chest radiography because our study focused on distinguishing whether nodules were calcified or not. Consequently, image reading of the present study was different from that of actual clinical situations.

In conclusion, the soft tissue image obtained by one-shot DES with a flat-panel detector has added value in distinguishing calcified from non-calcified nodules on chest radiographs compared to using standard images alone, especially by less experienced radiologists. Therefore, adding soft tissue images to the analysis may reduce the number of CT examinations for diagnosing calcification.

## Methods

### Ethics

The institutional review board of Niigata University approved this study and waived informed consent due to the observational and retrospective nature of the study. All methods were performed in accordance with the relevant guidelines and regulations. No funding was received for this study.

### Study population

Using radiological database, we searched patients who underwent chest radiography between January 2019 and March 2020 and whose radiology report included “nodule.” As a result, 688 patients were found. Of these, the inclusion criterion was patients who received CT performed within 3 months of one-shot DES chest radiography; CT was used as the reference standard for calcification and non-calcification. The exclusion criteria were patients with duplicate examination, nodules > 3 cm, patients with more than three nodules, and unclear nodules on chest radiographs. Finally, 155 nodules (48 calcified and 107 non-calcified; maximum diameter ≤ 3 cm) from 139 patients (77 men and 62 women; median age, 72 years) were analysed in this study. The selection and exclusion of the study population were first performed by a radiologist with 5 years of experience and were then confirmed by a radiologist with 18 years of experience; both radiologists did not participate in the evaluation of nodule calcification on chest radiography. Based on a previous study^1^, calcified nodules in the present study were defined as nodules containing diffuse or partial calcification, while non-calcified nodules were defined as nodules with no calcification at all.

### Image acquisition

All patients underwent posteroanterior chest radiography during deep inspiration in a standing position using one-shot DES with a flat-panel detector (FUJIFILM DR CALNEO Dual, Fujifilm) at a tube voltage of 120 kV and a tube current of 250 mA. This system consists of two types of X-ray detectors with different characteristics and can generate two images in a single X-ray exposure. Using the energy subtraction technique, soft tissue images were produced in addition to standard images. All CT examinations were performed using a multi-slice detector.

### Image analysis

Five radiologists with 26, 14, 8, 6, and 3 years of experience (readers 1 − 5), respectively, evaluated whether the nodules were calcified using chest radiography. Images were evaluated in two sessions at 4-week intervals: using the standard image alone and using both the standard and soft tissue images. In both sessions, the images were evaluated without referring to clinical information or CT images, but all the readers were given information on the location of the nodules on the chest radiography. The level of confidence for the presence of calcification was evaluated using the following rating scale: 1 = definitely calcified, 2 = probably calcified, 3 = equivocal, 4 = probably not calcified, and 5 = definitely not calcified. Rating scales of 1 and 2 were assigned to calcified nodules and rating scales of 3, 4, and 5 as non-calcified nodules. Subsequently, another two radiologists with 5 and 18 years of experiences who did not participate in the above-mentioned image reading (same radiologists who determined the study population) evaluated the following nodule characteristics using CT images (slice thickness, 1–2 mm) in a consensus manner: location, pattern of calcification (diffuse, central, laminated, popcorn, or other), type of non-calcified nodules (solid, part-solid, or pure ground-glass nodule), diameter, and percentage of solid component in part-solid nodules. They also assessed whether nodules overlapped with the bone or not using chest radiography. Image reading was performed using a picture archiving and communications system with a high-definition liquid crystal display monitor.

### Statistical analysis

The inter-observer agreement among the five readers was calculated using Fleiss’ kappa. According to Landis and Koch^18^, a kappa value of < 0.00 was interpreted as poor, 0.00 − 0.20 as slight, 0.21 − 0.40 as fair, 0.41 − 0.60 as moderate, 0.61 − 0.80 as substantial, and 0.81 − 1.00 as almost perfect. The sensitivity, specificity, accuracy, and AUC were calculated to determine the diagnostic performance. Moreover, the misdiagnosis ratios (false positive plus false negative ratios) were examined for nodules that overlapped with the bone and those that did not. The AUC was compared using the DeLong test^19^, and other diagnostic indices were compared using McNemar’s test. Statistical significance was set at p < 0.05. All statistical analyses were performed using SPSS (version 26, IBM) or R (version 4.04, R Core Team)^20^.

## Data Availability

The data used in this study are available from the corresponding author upon reasonable request.

## Abbreviations

AI: Artificial intelligence
AUC: Area under the receiver operating characteristic curve
DES: Dual-energy subtraction
